# Comparative evaluation of 14 *hlyA*-based loop-mediated isothermal amplification assays for the rapid detection of *Listeria monocytogenes*

**DOI:** 10.3389/fmicb.2026.1913307

**Published:** 2026-08-28

**Authors:** Leticia Mallmann, Beilei Ge, Madelyn Springer, Kelly J. Domesle, Vanessa de Vasconcellos, Yi Chen, Eduardo Ximenes

**Affiliations:** 1 Department of Environmental and Occupational Health, School of Public Health, Indiana University, Bloomington, IN, United States; 2 Office of Applied Science, Center for Veterinary Medicine, U.S. Food and Drug Administration, Laurel, MD, United States; 3 Office of Applied Microbiology and Technology, Human Foods Program, U.S. Food and Drug Administration, College Park, MD, United States

**Keywords:** comparative evaluation, *hlyA*, *Listeria monocytogenes*, loop-mediated isothermal amplification, sensitivity, specificity, quantification capability

## Abstract

**Introduction:**

Rapid detection of *Listeria monocytogenes* in food is critical for implementing timely measures to reduce foodborne listeriosis and subsequent fatalities. While multiple *L. monocytogenes* loop-mediated isothermal amplification (LAMP) assays targeting the *hlyA* gene (listeriolysin O) have been reported in the literature, no independent comparative evaluation has been conducted.

**Methods:**

In this work, we evaluated the performance of 14 *hlyA*-based *L. monocytogenes* LAMP assays, using an FDA-developed *L. monocytogenes* LAMP assay targeting the same gene for benchmarking. All assays were performed in an enclosed OptiGene Genie II platform using a harmonized protocol (not individually reported/optimized conditions). The assay sensitivity (limit of detection, LOD) and specificity (inclusivity/exclusivity) were determined using a ten-fold serially diluted reference *L. monocytogenes* culture (ATCC 19115), and a panel of 50 *L. monocytogenes* strains, 20 other *Listeria* spp. strains, and 31 non-*Listeria* strains, respectively. We also evaluated the quantification capabilities of these LAMP assays.

**Results and Discussion:**

The LODs for these 14 LAMP assays using *L. monocytogenes* ATCC 19115 ranged from exceeding 10^6^ cells per reaction (i.e., nondetectable for three of them at the highest concentration tested) to 10^2^ cells per reaction, with the latter including the FDA LAMP assay. The inclusivity ranged from 20 to 90% detection using the initial templates prepared at approximately 10^0^-10^2^ cells per reaction whereas 100% inclusivity was observed for two selected and the FDA LAMP assays at higher template concentrations. All 14 assays had 100% exclusivity. The quantification capabilities of these assays varied with correlation coefficients ranging from 0.681 to 0.992. This comprehensive evaluation of 14 *hlyA*-based *L. monocytogenes* LAMP assays highlights the critical role of assay design in enabling rapid, sensitive, and specific detection of this prominent foodborne pathogen to ensure food safety and protect public health.

## Introduction

*Listeria monocytogenes* is a G-positive facultative intracellular pathogen widely distributed in natural and built environments (Orsi and Wiedmann, 2016). It can grow and form biofilms under low refrigerating temperatures, thus persisting in a variety of foods and food production surfaces (Chowdhury and Anand, 2023; Poutou-Piñales et al., 2026). Foodborne infections with *L. monocytogenes* result mainly due to contamination from a variety of food sources (Acciari et al., 2015; Avila-Novoa et al., 2024; Huss et al., 2000; Lokerse et al., 2016). Ready-to-eat products and fresh produce are two of the main sources of listeriosis outbreaks, accounting for hundreds of hospitalizations and high fatality rates for high-risk individuals [CDC, 2025, EFSA (European Food Safety Authority) and ECDC (European Centre for Disease Prevention and Control), 2021, FDA, 2026; Jackson et al., 2018; Raheem, 2016]. Thus, rapid and highly sensitive methods for detecting *L. monocytogenes* in foods are a critical need in food safety.

Protocols such as the FDA's *Bacteriological Analytical Manual* (BAM) Chapter 10 (Hitchins et al., 2022) recommend culture-based isolation and identification followed by real-time polymerase chain reaction (PCR) confirmation. Alternatively, commercially available rapid test kits have been developed and adopted by regulatory agencies (Andrews and Hammack, 2023; USDA, 2024). These methods face limitations due to high cost of equipment, complexity, and high susceptibility to inhibitory compounds in food. Loop-mediated isothermal amplification (LAMP) is a rapid nucleic acid amplification technology that offers a promising alternative to PCR for fast, robust, and cost-effective pathogen detection. LAMP has gained wide application in detecting several bacterial pathogens from human and animal foods, including *Salmonella* (Domesle et al., 2018; Hara-Kudo et al., 2005), *Campylobacter* (Kreitlow et al., 2021; Yamazaki et al., 2009), Shiga toxin-producing *Escherichia coli* (Wang et al., 2012a, 2014), *Vibrio* (Hu et al., 2021; Tian et al., 2022), and others. LAMP employs a strand-displacing DNA polymerase and a set of four to six (two outer, two inner, and no loop or one to two loop) primers to generate up to 10^9^ target DNA copies within an hour of isothermal amplification at 60–65°C (Nagamine et al., 2002; Notomi et al., 2000). LAMP assays are less susceptible to inhibitory substances, such as particles and compounds present in food matrices (Kaneko et al., 2007; Yang et al., 2014), highlighting its applicability in food testing (Yang et al., 2018).

Several nucleic acid amplification methods (i.e., PCR, real-time PCR, and LAMP) have been reported for the detection of *L. monocytogenes* primarily targeting specific virulence-related genes. These included listeriolysin O (*hlyA*), phospholipase C (*plcA, plcB*), internalin (*inlA, inlB*), and actin polymerization protein (*actA*; Chen et al., 2017; Liu, 2013). The *hlyA* gene is highly conserved in *L. monocytogenes*, and therefore the most common target for designing LAMP assays. It encodes a cholesterol-dependent pore-forming cytolysin that is crucial for the survival of Gram-positive pathogens during the hemolytic infection (Cossart et al., 1989; Nguyen et al., 2019). Multiple LAMP assays have been developed for the detection of *L. monocytogenes* targeting various regions of the *hlyA* gene (Cheng et al., 2016; Garrido-Maestu et al., 2018; Huang et al., 2015; Ma et al., 2019; Pisamayarom et al., 2017; Rivas-Macho et al., 2023; Shan et al., 2012; Tang et al., 2011; Wan et al., 2012; Wang et al., 2011, 2012b; Ye et al., 2015); however, independent comparative evaluation has not been conducted.

In the present study, we comparatively evaluated 14 *hlyA*-based *L. monocytogenes* LAMP primer sets (assays) for sensitivity (limit of detection, LOD), specificity (inclusivity/exclusivity), and quantification capability. Among these assays, a new *L. monocytogenes* LAMP assay targeting the same gene developed at the FDA's Center for Veterinary Medicine (CVM), referred to as the FDA-2026 assay hereafter (Ge et al., 2026), was used for benchmarking.

## Materials and methods

### Bacterial strains

Fifty *L. monocytogenes* strains, 20 other *Listeria* spp. strains (*L. grayi, L. innocua, L. ivanovii, L. seeligeri*, and *L. welshimeri*), and 31 non-*Listeria* strains (*Bacillus, Bifidobacterium, Citrobacter, Enterococcus, Escherichia, Klebsiella, Lacticaseibacillus, Salmonella, Serratia, Shigella, Staphylococcus, Streptococcus*, and *Yersinia*) were tested for specificity comparative evaluation, using the same strain list as described previously (Ge et al., 2026). A reference *L. monocytogenes* strain ATCC 19115 (Manassas, VA, USA) was selected as the positive control and used in sensitivity comparative evaluation. All bacterial strains were cryopreserved at −80°C in brain heart infusion (BHI) broth (BD, Sparks, MD, USA) with 20% glycerol and cultured onto blood agar plates (BAP; Thermo Fisher Scientific, Waltham, MA, USA) at 37°C overnight.

### Preparation of DNA templates

All bacterial DNA templates were prepared following the Association of Official Analytical Collaboration (AOAC) and INTERNATIONAL FDA guidelines (AOAC, 2023; FDA, 2019a,b). Single colonies on BAP were transferred to 5 mL of BHI broth and incubated at 37°C overnight with agitation. For *L. monocytogenes* strains, overnight cultures were serially diluted in 0.1% (w/v) peptone water to *ca*. 10^3^-10^5^ CFU/mL (this range was due to different overnight culture concentrations). For other *Listeria* spp. strains, overnight cultures were used directly without further dilution per AOAC guideline (AOAC, 2023). DNA extraction was performed with BioSprint 96 One-For-All Vet Kit (INDICAL Inc., Orlando, FL, USA) on a KingFisher Flex-96 Purification System (Thermo Fisher Scientific). DNA extracts were stored at −20°C for up to 24 months prior to analyses.

### Preparation of LAMP primer mixes

**Figure 1:**
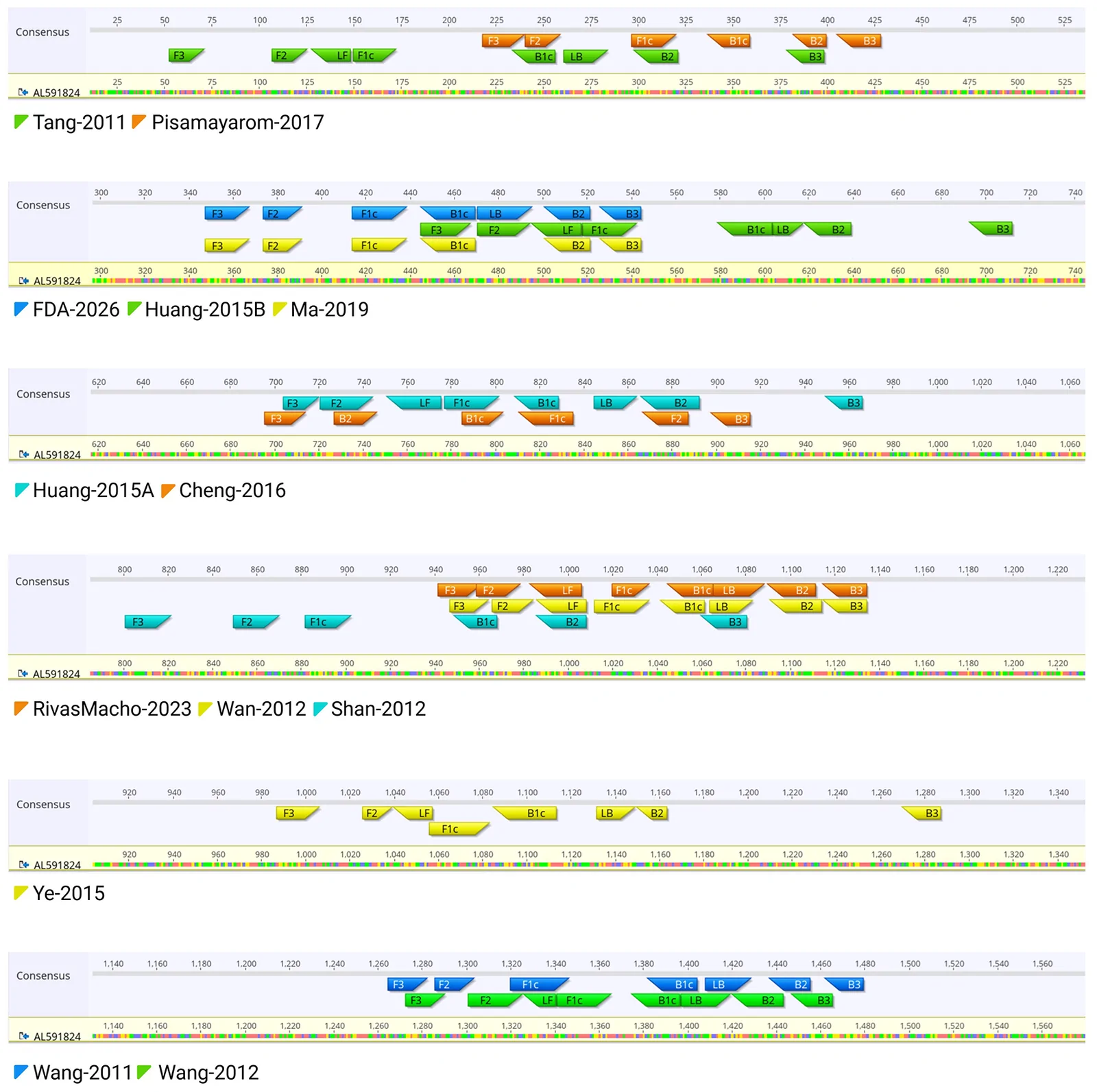
Sequence alignments illustrating positions of the 14 *Listeria monocytogenes hlyA*-based loop-mediated isothermal amplification (LAMP) primer sets (assays) evaluated in the study against the reference *L. monocytogenes* EGD-e complete genome (GenBank accession no. AL591824) using Geneious Prime (2026.0.2). Primer set Garrido-Maestu-2018 could not be mapped due to high frequency of mismatches. Primer sets targeting similar regions are grouped together. For each primer set, F3 is the forward outer primer, B3 is the backward outer primer, FIP (forward inner primer) consists of a complementary sequence of F1 (F1c) and a sense sequence of F2. BIP (backward inner primer) consists of a complementary sequence of B1 (B1c) and a sense sequence of B2. LF is the loop forward primer, and LB is the loop backward primer.

Sequences of the previously described LAMP primer sets (Table 1) were retrieved from respective studies (Cheng et al., 2016; Garrido-Maestu et al., 2018; Huang et al., 2015; Ma et al., 2019; Pisamayarom et al., 2017; Rivas-Macho et al., 2023; Shan et al., 2012; Tang et al., 2011; Wan et al., 2012; Wang et al., 2011, 2012b; Ye et al., 2015) and from the FDA/CVM study (Ge et al., 2026). The primer locations on the target *hlyA* gene are visualized in Figure 1. All primers were ordered from Integrated DNA Technologies (IDT; Coralville, IA, USA) and resuspended in nuclease-free water for a final concentration of 100 μM. Primer mixes (10 × ) were prepared to follow the final concentrations in the LAMP reaction mix (below) as the optimized ratio for the FDA-2026 assay, that is, 0.1 μM F3 and B3, 1.8 μM FIP and BIP, and 1 μM LF and/or LB (Ge et al., 2026).

**Table 1:** Sequences of 14 *Listeria monocytogenes hlyA*-based loop-mediated isothermal amplification (LAMP) primer sets (assays) evaluated in the present study.

Primer set	Name	Sequence (5^′^-3^′^)	References
FDA-2026	F3	AGTTGTGAATGCAATTTCGA	Ge et al., 2026
	B3	CTGCGTTGTTAACGTTTGA	
	FIP	AGGGAGAACATCTGGTTGATTTTCTCCTATCCAGGTGCTCTCG	
	BIP	CGTGATTCATTAACACTCAGCATTGGTGGCATTTTTTACAACGATT	
	LB	TTTGCCAGGTATGACTAATCAAGAC	
Tang-2011	F3	TTGCGCAACAAACTGAAGC	Tang et al., 2011
	B3	GCTTTTACGAGAGCACCTGG	
	FIP	CGTGTTTCTTTTCGATTGGCGTCTTTTTTTCATCCATGGCACCACC	
	BIP	CCACGGAGATGCAGTGACAAATGTTTTGGATTTCTTCTTTTTCTCCACAAC	
	LF	TAGGACTTGCAGGCGGAGATG	
	LB	GCCAAGAAAAGGTTACAAAGATGG	
Wang-2011	F3	ATCACTCTGGAGGCTACG	Wang et al., 2011
	B3	TTCTCCACCATTCCCAAG	
	FIP	TGAACAATTTCGTTACCTTCAGGATTGCTCAATTCAACATCTCTT	
	BIP	TAGCTCATTTCACATCGTCCATCAGTGCATTCTTTGGCGTAA	
	LB	TTGCCAGGTAACGCAAGAAAT	
Shan-2012	F3	CAGATTTTTCGGCAAAGCTGT	Shan et al., 2012
	B3	TGAACTTCATCTTTTGCGGAG	
	FIP	TGACGGCCATATGCCACACGAGTGAATGCAGAAAATCCTCCT	
	BIP	GCTGCTTTTGACGCTGCCGTATTTGTCAGTTCTACATCACCTG	
Wan-2012	F3	AAGCTGCTTTTGATGCTG	Wan et al., 2012
	B3	TCGATTAAAAGTAGCGCCTT	
	FIP	CGGCTTTGAAGGAAGAATTTTTGATCGTAAGCGGAAAATCTGTC	
	BIP	TACGGAGGTTCCGCAAAAGATTTTCAAAATATCGCGTAAGTCTC	
	LF	ATTTGTTAGTTCTACATCACCTG	
	LB	AAGTTCAAATCATCGACGGC	
Wang-2012	F3	GGAGGMTACGTTGCTCAA	Wang et al., 2012b
	B3	AAGCTAAACCAGTGCATTC	
	FIP	TCGCTCCAGTTTTTATGTTGAACACCTTGGGATGAARTAAATTATGATCC	
	BIP	AGCAAGCTAGCTCATTTCACATAGCGTAAACATTAATATTTCTCGC	
	LF	ACTTCCATTKCTTTA	
	LB	CGTCCATCTATTTGCCAGGTAAC	
Huang-2015	F3	ACTTCGGCGCAATCAGT	Huang et al., 2015
	B3	GGCAGCATCAAAAGCAG	
	FIP	GGAAGGTCTTGTAGGTTCATTAACAGAAGGGAAAATGCAAGAAGAAGTC	
	BIP	TCGGCAAAGCTGTTACTAAAGAGCCACACTTGAGATATATGCAGGAG	
	LF	TCACGTTATAGTAAATTTGTTTAAA	
	LB	TTGGAGTGAATGCAGAAAAT	
Huang-2015	F3	CGTGATTCATTAACACTTAGCAT	Huang et al., 2015
	B3	CGCCGAAGTTTACATTCAAG	
	FIP	GCGTTGTTAACGTTCGATTTAGTAGTTTGCCAGGAATGACTAATCAAGA	
	BIP	TGCTCAAGCTTATCCAAATGTAAGTACTGTAAGCCATTTCGTCATCA	
	LF	CATTTTTTACAACGATTTTATTG	
	LB	GCAAAAATTGATTAT	
Ye-2015	F3	AGGTCATGTAGGACTGAACA	Ye et al., 2015
	B3	TGCAACGTATCCTCCAGA	
	FIP	ACCGTCGATGATTTGAACTTCATTCTTCCTTCAAAGCCGTAA	
	BIP	ACCTCGGAGACTTACGAGATATGTTTGTTGTATAGGCAATGGG	
	LF	TTGCGGAGCCACCGTAAA	
	LB	CGGGAAACACCAGGAGTT	
Cheng-2016	F3	TTTCAAATAAACTTGACGGC	Cheng et al., 2016
	B3	AATGTAAACTTCGGCGCA	
	FIP	GGCAAAGCTGTTACTAAAGAGCAGTTTTTACACTTGAGATATATGCAGGA	
	BIP	CTGGAAGGTCTTGTAGGTTTTTTAAAATGCAAGAAGAAGTCAT	
Pisamayarom-2017	F3	ACAATGTATTAGTATACCACGGA	Pisamayarom et al., 2017
	B3	TCTGGTTGATTTTCTACTAATTCC	
	FIP	GGATTTCTCTTTTTCTCCACAACGTTTTGATGCAGTGACAAATGTGC	
	BIP	GCAGACATCCAAGTTGTAAATGCTTTTCGCTTTTACGAGAGCACC	
Garrido-Maestu-2018	F3	TGTGTTTGAGCTAGTGGTTTGG	Garrido-Maestu et al., 2018
	B3	CCCATTAGGCGGAAAAGCATAT	
	FIP	GCAGCGCTCTCTATACCAGGTACAttttAATGTCCATGTTATGTCTCCGTTA	
	BIP	AGGTTTGTTGTGTCAGGTAGAGCGttttCGCTTAATAACTGGAATAAGCCAA	
	LB	CATCCATTGTTTTGTAGTTACAGAG	
Ma-2019	F3	AGTTGTGAATGCAATTTCGA	Ma et al., 2019
	B3	CTGCGTTGTTAACGTTTGA	
	FIP	AGGGAGAACATCTGGTTGATTTTCTCCTATCCAGGTGCTCTCG	
	BIP	CGTGATTCATTAACACTCAGCATTGGTGGCATTTTTTACAACGATT	
Rivas-Macho-2023	F3	AGTAAAAGCTGCTTTTGAYG	Rivas-Macho et al., 2023
	B3	YCGRTTAAAAGTAGCRCCTT	
	FIP	CGGCTTTGAAGGAAGAATTTTTGATCTGCCGTAAGYGGRAAAT	
	BIP	GGWGGYTCCGCAAAAGATGATTTCAAAATATCKCGTAAGTCTCC	
	LF	TTGTYAGTTCTACATCACCTGAGA	
	LB	AGTTCAAATCATCGACGGYAACCT	

### LAMP assays

A harmonized protocol was used. Briefly, the LAMP assay reaction mix of total volume of 25 μL contained 15 μL of GspSSD2.0 Isothermal Mastermix (ISO-004; OptiGene Ltd., West Sussex, UK), 2.5 μL of each 10 × primer mix (IDT), 5 μL of each DNA template, and 2.5 μL of nuclease-free water into Genie 8-well strips (OptiGene Ltd.). LAMP reactions occurred in a Genie II closed-tube platform (OptiGene Ltd.) with real-time fluorescence detection. The isothermal amplification phase was set at 65°C for 30 min, followed by an anneal phase from 98°C to 80°C, at 0.05°C/s decrement. The anneal phase of the LAMP assay using the Huang-2015 primer set was further extended to 76°C (instead of 80°C) to observe a recordable anneal peak at lower than 80°C (the default instrument setting). DNA extracted from the reference strain *L. monocytogenes* ATCC 19115 at *ca*. 10^6^ cells/reaction was used as the positive control (PC) and nuclease-free water as the no-template control (NTC) in each run. Results were called through real-time fluorescence reading at the parameters of time-to-positivity (peak ratio—T_p_), in min, and annealing temperature of amplicons (anneal peak—T_a_), in °C. T_p_ was obtained when the maximum amplification rate curve was reached, while T_a_ was obtained at the maximum value of the anneal derivative curve. In the present evaluation study, samples were considered LAMP positive using a standard T_p_ cutoff value of 20 min for Genie II with the ISO-004 kit and recordable T_a_. This was determined as a conservative measure to ensure specific amplifications and avoid low-confidence late signals, similar to that used for a *Salmonella enterica* LAMP assay (Andrews et al., 2026).

### Specificity, sensitivity, and quantification capability

Specificity of the 14 LAMP primer sets were comparatively evaluated using an inclusivity panel (*n* = 50) and an exclusivity panel (*n* = 51). Inclusivity templates were used at approximately 10^0^-10^2^ cells/reaction initially, followed by higher concentrations for selected primer sets. Exclusivity templates were used at approximately 10^6^ cells/reaction. Sensitivity (LOD) comparative evaluations were performed in triplicate using serially diluted DNA templates of *L. monocytogenes* ATCC 19115 from 2 × 10^8^ to 2 × 10^2^ cells/mL (*ca*. from 10^6^ to 10^0^ cells/reaction). Quantification capability (linearity) of the assays was comparatively evaluated using sensitivity testing results on LAMP-positive sample series.

### Statistical analysis

Triplicate T_p_ and T_a_ values were summarized as means and standard deviations in Excel (Microsoft 360, version 2025). Probability of detection (POD) was analyzed using McNemar's test on paired strain level detection outcomes, with Benjamini–Hochberg false discovery rate adjustment. Effects of primer set and DNA concentration on T_p_ and T_a_ were assessed by two-way ANOVA followed by Tukey-adjusted estimated marginal means. Quantification capability among primer sets was compared using *R*^2^ values. Analyses were performed in R software (version 4.5.2) with statistical significance at *p* < 0.05.

## Results

### Specificity

None of the 14 *hlyA*-based LAMP assays (13 primer sets from the literature and the FDA-developed *L. monocytogenes* LAMP assay for benchmarking) produced false-positive results (recordable T_p_ and T_a_) for the 51 exclusivity templates evaluated at the growth limit of the organisms (data not shown). Using an extensive collection of 50 *L. monocytogenes* initial inclusivity templates at approximately 10^0^-10^2^ cells/reaction, inclusivity of the 14 LAMP assays varied, with average T_p_ values ranging from 9.1 to 18.7 min and average T_a_ values between 80.7 and 83.6°C (Table 2). Three primer sets (Huang-2015, FDA-2026, and Rivas-Macho-2023) showed the highest performance, detecting at least 80% of the 50 *L. monocytogenes* initial templates. Further testing of Huang-2015 and Rivas-Macho-2023 primer sets with higher template concentrations showed 100% inclusivity for both, with positive detection of various templates ranging from ~10^1^ to 10^5^ cells/reaction (data not shown). A similar result was observed for the FDA-2026 assay (Ge et al., 2026). Over half of *L. monocytogenes* strains were detected by primer sets reported in Wang-2011, Huang-2015, Wan-2012, and Ma-2019 studies. In contrast, none of the initial inclusivity templates were detected by primer sets reported in Tang-2011, Shan-2012, and Wang-2012 studies. T_a_ signals were recorded for the Shan-2012 primer set for 22 strains (82.8 ± 0.1°C), but no T_p_ was observed (Table 2).

**Table 2:** Probabilities of detection (POD), confidence intervals, and average T_p_/T_a_ values of 14 *Listeria monocytogenes hlyA*-based loop-mediated isothermal amplification (LAMP) primer sets (assays) for comparative inclusivity evaluation of 50 *L. monocytogenes* strains at initial DNA template concentrations of approximately 10^0^-10^2^ cells/reaction.

Primer set ID	POD (%)	CI (95%)	Average T_p_ (min)	Average T_a_ (°C)
FDA-2026^a^	86	70–90	10.5 ± 2.8	82.5 ± 0.3
Tang-2011	0	NA	NA	NA
Wang-2011	72	60–80	14.0 ± 2.9	82.0 ± 0.2
Shan-2012	0	NA	NA	82.8 ± 0.1
Wan-2012	60	50–70	9.1 ± 3.8	83.6 ± 1.7
Wang-2012	0	NA	NA	NA
Huang-2015^a^	90	80–100	11.5 ± 1.9	83.0 ± 0.1
Huang-2015	68	50–80	16.8 ± 2.1	80.7 ± 0.1
Ye-2015	48	30–60	9.4 ± 2.4	83.6 ± 0.2
Cheng-2016	20	10–30	18.7 ± 1.4	82.2 ± 0.1
Pisamayarom-2017	30	20–40	15.9 ± 3.5	82.1 ± 0.1
Garrido-Maestu-2018	26	10–40	17.2 ± 1.7	83.1 ± 0.2
Ma-2019	52	40–70	13.0 ± 2.8	82.6 ± 0.5
Rivas-Macho-2023^a^	80	70–90	14.2 ± 1.5	83.3 ± 0.1

^a^Further testing of these primer sets with serial dilutions resulted in an POD of 100%.

Statistical analysis confirmed that LAMP primer sets Huang-2015, FDA-2026, and Rivas-Macho-2023 had superior PODs than primer sets Cheng-2016, Garrido-Maestu-2018, and Pisamayarom-2017 (*p* < 0.001). No significant differences were observed within higher- and lower-performing groups (*p* > 0.05). Primer sets Wang-2011, Huang-2015, Wan-2012, Ma-2019, and Ye-2015 showed intermediate and significantly lower PODs than higher-performing sets (*p* < 0.05). No significant differences were observed within the intermediate performing group (*p* > 0.05).

### Sensitivity

**Figure 2:**
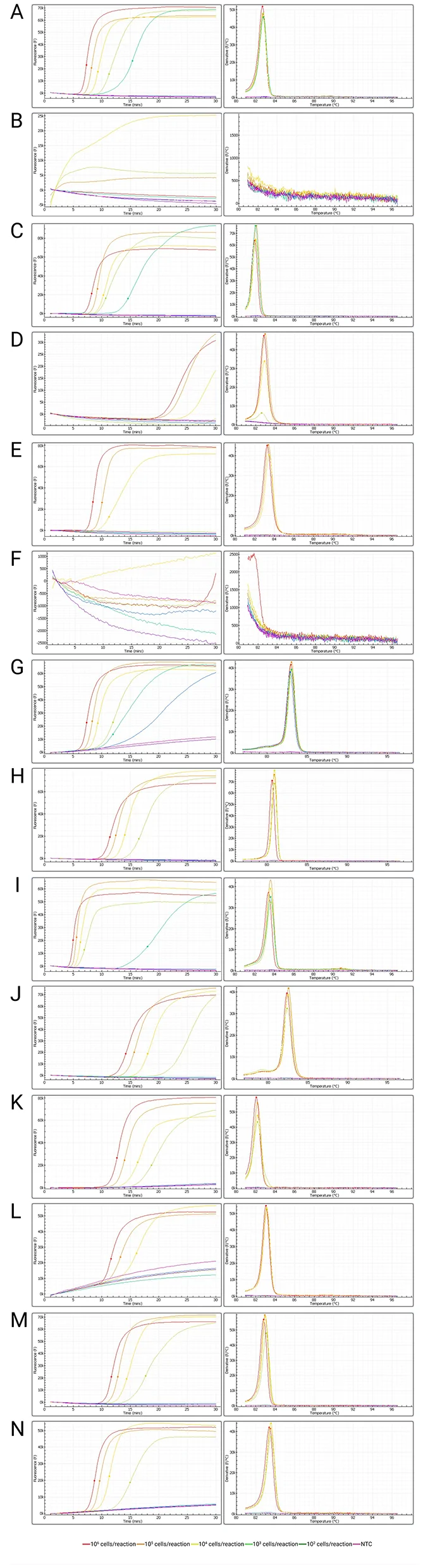
Representative amplification graphs and associated anneal derivative curves of the 14 *hlyA*-based *Listeria monocytogenes* loop-mediated isothermal amplification (LAMP) primer sets (assays) when testing the reference *L. monocytogenes* ATCC 19115 template series from 10^6^ cells/reaction to 1 cell/reaction. **(A)** FDA-2026, **(B)** Tang-2011, **(C)** Wang-2011, **(D)** Shan-2012, **(E)** Wan-2012, **(F)** Wang-2012, **(G)** Huang-2015, **(H)** Huang-2015, **(I)** Ye-2015, **(J)** Cheng-2016, **(K)** Pisamayarom-2017, **(L)** Garrido-Maestu-2018, **(M)** Ma-2019, and **(N)** Rivas-Macho-2023. Samples 1–7 are serially diluted *L. monocytogenes* ATCC 19115 templates at 10^6^ cells/reaction (red), 10^5^ cells/reaction (orange), 10^4^ cells/reaction (yellow), 10^3^ cells/reaction (light green),10^2^ cells/reaction (green), 10^1^ cells/reaction (blue), 1 cell/reaction (purple), and NTC being nuclease-free water (magenta).

**Figure 3:**
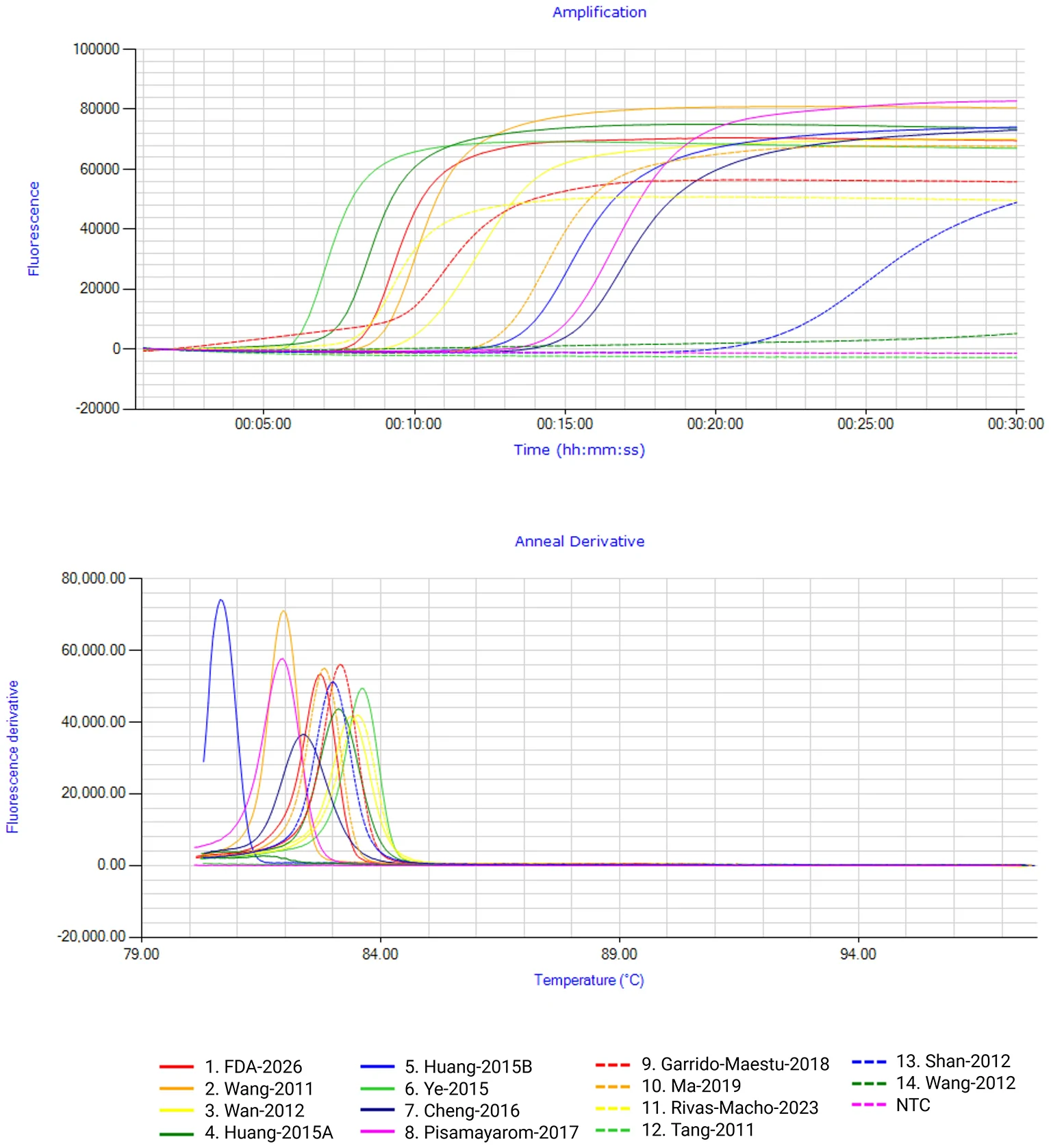
Amplification graph and associated anneal derivative curve for the 14 *Listeria monocytogenes hlyA*-based loop-mediated isothermal amplification (LAMP) primer sets (assays) analyzing *L. monocytogenes* ATCC 19115 template at 10^4^ cells/reaction. Those with valid amplifications included (1) FDA-2026, (2) Wang-2011, (3) Wan-2012, (4) Huang-2015, (5) Huang-2015, (6) Ye-2015, (7) Cheng-2016, (8) Pisamayarom-2017, (9) Garrido-Maestu-2018, (10) Ma-2019, and (11) Rivas-Macho-2023, and those without valid amplifications included (12) Tang-2011, (13) Shan-2012, and (14) Wang-2012. NTC (no-template control) is nuclease-free water.

Three of the 14 LAMP primer sets (Tang-2011, Shan-2012, Wang-2012) could not produce positive results, even at the highest concentration. Using the reference *L. monocytogenes* ATCC 19115 template series from 10^6^ cells/reaction to 1 cell/reaction (Figure 2), the Shan-2012 set generated recordable T_a_ values from 10^6^ cells/reaction to 10^3^ cells/reaction but no T_p_. Six primer sets (FDA-2026, Wang-2011, Wan-2012, Ye-2015, Huang-2015, and Rivas-Macho-2023) were able to generate T_p_ within 10 min at DNA concentrations of 10^6^ and 10^5^ cells/reaction. FDA-2026, Ye-2015, and Huang-2015 also generated T_p_ within 10 min at 10^4^ cells/reaction and Ye-2015 at 10^3^ cells/reaction. The FDA-2026 assay reached the lowest LOD among all primer sets, with triplicate positive results at 10^2^ cells/reaction. T_a_ values observed in sensitivity assays averaged around 82°C. Figure 3 shows an amplification graph and associated anneal derivative curve of a LAMP run that included all 14 LAMP primer sets using *L. monocytogenes* ATCC 19115 template at 10^4^ cells/reaction. The relative rapidity of the assays is apparent with the top three performers being Ye-2015, Huang-2015, and FDA-2026.

Two-way ANOVA revealed strong effects of primer set and DNA concentration on T_p_ values, as well as the interaction of both factors (*p* < 0.0001). Stronger effects of primer set alone and in interaction with DNA concentration were observed for T_a_ (*p* < 0.001) when compared to DNA concentration alone (*p* = 0.04). Since residual diagnostics indicated deviations from normality (Shapiro–Wilk test) and heterogeneity of variance (Levene's test), data was log-transformed, yielding results consistent with those from untransformed data. However, because homogeneity of variance was only met for log-transformed T_a_ values, but not for T_p_ values, Tukey-adjusted *post-hoc* comparisons were performed to compare primer sets within each DNA concentration. These revealed significant differences in T_p_ values across specific primer sets. FDA-2026, Huang-2015, and Ye-2015 showed substantial lower values (indicating faster reactions) at 10^6^-10^4^ cells/reaction compared to other primer sets (*p* < 0.001). Primer set Ye-2015 showed significant higher T_a_ values compared to others at 10^6^ and 10^5^ cells/reaction (*p* < 0.0001). No significant diferences among T_a_ values were revealed for other primer sets (*p* > 0.05).

### Quantification capability

**Figure 4:**
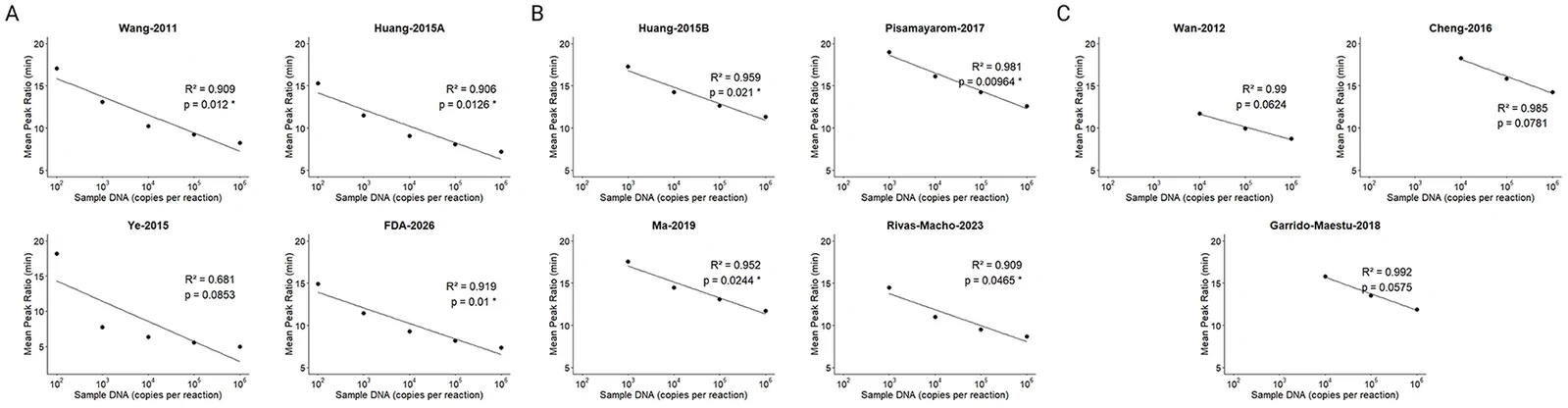
Quantification capability of the 14 *Listeria monocytogenes hlyA*-based loop-mediated isothermal amplification (LAMP) primer sets (assays) when testing the reference *L. monocytogenes* ATCC 19115 template series from 10^6^ cells/reaction to 1 cell/reaction. Correlation was assessed between DNA template concentration and peak ratio (T_p_). **(A)** Assays (*n* = 4) with quantification down to 10^2^ cells/reaction. **(B)** Assays (*n* = 4) with quantification down to 10^3^ cells/reaction. **(C)** Assays (*n* = 3) with quantification down to 10^4^ cells/reaction. Three primer sets (Tang-2011, Shan-2012, and Wang-2012; not shown) did not have valid amplifications (T_p_) at any of the levels.

Strong linearity between DNA concentration (from 10^6^ cells/reaction to 10^5^, 10^4^, 10^3^, or 10^2^ cells/reaction) and T_p_ was observed for LAMP assays using primer sets Pisamayarom-2017 (*R*^2^ = 0.981; *p* = 0.009), Huang-2015 (*R*^2^ = 0.959, *p* = 0.021), Ma-2019 (*R*^2^ = 0.952, *p* = 0.024), FDA-2026 (*R*^2^ = 0.919, *p* = 0.010), Huang-2015 (*R*^2^ = 0.906; *p* = 0.0126), and Rivas-Macho-2023 (*R*^2^ = 0.909, *p* = 0.046; Figure 4). Two sets of four assays (Wang-2011, Huang-2015A, Ye-2015, and FDA-2026; and Huang-2015B, Pisamayarom-2017, Ma-2019, and Rivas-Macho-2023) were quantitative down to 10^2^ cells/reaction and 10^3^ cells/reaction, respectively (Figures 4A, B). These tested primers showed significant quantification capability, except for the assay with primer set Ye-2015 (*p* = 0.0853). Linearity obtained for all three assays (Wan-2012, Cheng-2016, and Garrido-Maestu-2018) that were quantitative down to 10^4^ cells/reaction was not statistically significant (*p* > 0.05; Figure 4C). No significant association was obtained for any assays between DNA concentration and T_a_ values (*p* > 0.05).

## Discussion

All 14 *hlyA*-based *L. monocytogenes* assays had 100% exclusivity, in agreement with original reports (Cheng et al., 2016; Garrido-Maestu et al., 2018; Ge et al., 2026; Huang et al., 2015; Ma et al., 2019; Pisamayarom et al., 2017; Rivas-Macho et al., 2023; Shan et al., 2012; Tang et al., 2011; Wan et al., 2012; Wang et al., 2011, 2012b; Ye et al., 2015). Notably, this present study used the largest number of exclusivity strains (*n* = 51) among all studies referenced here.

The 14 LAMP assays reported 100% inclusivity in their original reports with the number of *L. monocytogenes* strains tested ranging from 1 to 92 except the Wang-2012 primer set which tested 182 food isolates and reported 96.7% inclusivity (Wang et al., 2012b). The inclusivity of these assays in detecting 50 *L. monocytogenes* strains (of various serotypes and lineages) using the initial templates prepared at *ca*. 10^0^-10^2^ cells/reaction differed significantly under the parameters in the present study (Table 2). Assays with primer sets Huang-2015, FDA-2026, and Rivas-Macho-2023 had the highest PODs (80%−90%). Further testing using templates at higher concentrations (up to 10^5^ cells/reaction) resulted in 100% inclusivity (Ge et al., 2026). Assays with primer sets Tang-2011, Shan-2012, and Wang-2012 could not detect any *L. monocytogenes* strains evaluated in the present study, despite previously reported to detect 12 (Tang et al., 2011) and 92 reference strains (Shan et al., 2012), and 176 food isolates (Wang et al., 2012b). All other primer sets were able to detect a higher number of *L. monocytogenes* strains than previously reported, owing to the extensive inclusivity panel used in the present study (*n* = 50). Further evaluation using templates at higher concentrations performed for Huang-2015, FDA-2026, and Rivas-Macho-2023 primer sets confirmed the 100% inclusivity of these top-performing assays.

Most original reports used *L. monocytogenes* strains with unspecified serotypes/lineages, making direct comparisons impossible. Other than primer sets Cheng-2016 and FDA-2026 (Cheng et al., 2016; Ge et al., 2026), none of the other 12 LAMP assays specified inclusivity template tested concentrations. It is hypothesized that similar concentrations as exclusivity, i.e., growth limits of the organisms, were used, in contrast to the recommended 10–100 fold of LODs (AOAC, 2023). Such high concentrations would not provide sufficient stringency for inclusivity evaluation, possibly resulting in overestimations. The effect of inclusivity template concentration on POD was clearly demonstrated in the present study with the expanded evaluation of primer sets Huang-2015 and Rivas-Macho-2023, corroborating findings reported for the FDA-2026 assay (Ge et al., 2026).

The sensitivity (LOD) observed for 14 LAMP assays differed significantly across primer sets (Figures 2, 3) and were generally lower than those originally reported, ranging from 1 CFU/reaction (Huang et al., 2015; Ma et al., 2019) to 3.7 × 10^3^ CFU/ml (Ye et al., 2015). Four of the LAMP primer sets reported dynamic ranges covering 3–8 log orders (down to 10^2^ cells/reaction) with *R*^2^ between 0.991 and 0.994 (Cheng et al., 2016; Garrido-Maestu et al., 2018; Rivas-Macho et al., 2023; Shan et al., 2012). In the present study, quantification down to 10^2^ cells/reaction was also observed for four primer sets (Wang-2011, Huang-2015A, Ye-2015, and FDA-2026), other than the four previously reported (Figure 4A).

Among parameters influencing LAMP performance, primer design, reaction components, reaction conditions, and template concentrations play essential roles. As shown in Figure 1, the 14 LAMP primer sets (some with one or two loop primers) targeted different regions of the target DNA sequence, where complementarity is crucial for amplification efficiency. This complementarity is strongly affected by the nucleotide polymorphism across target strains, which reduces the thermal stability of the primer-template duplex and consequently the amplification efficiency of molecular assays (Stadhouders et al., 2010; Vogels et al., 2020). Since LAMP recognizes up to eight regions within the target gene to ensure high specificity, the effect of mismatches should be evaluated carefully to identify highly conserved regions. Designing degenerate primers may be a viable option to accommodate nucleotide polymorphisms in given target regions, which was used previously (Rivas-Macho et al., 2023; Wang et al., 2012b). The use of loop primers was demonstrated to greatly accelerate the LAMP amplification (Nagamine et al., 2002). All the 14 LAMP assays, except Shan-2012, Cheng-2016, Pisamayarom-2017 and Ma-2019, have adopted one or two loop primers (Figure 1, Table 1). Primer set Ma-2019 had the same outer and inner primers as FDA-2026. By adding a loop primer (LB) in the latter, the FDA-2026 assay had 10 × higher sensitivity (Figure 2). This also resulted in much better tested inclusivity using 50 *L. monocytogenes* initial templates prepared at estimated 10^0^ to 10^2^ cells/reaction (Table 2), and better quantification capability (Figure 4). Surprisingly, primer set Garrido-Maestu-2018 (Garrido-Maestu et al., 2018) could not map to the reference, and it actually targeted the *plcA* gene, which was immediate upstream of *hlyA* (personal communication with Garrido-Maestu, A.).

The composition of LAMP reaction mixes can greatly affect LAMP amplification efficiency (Zhou et al., 2014). In earlier reports, 4–8 mM MgSO^4^ and 0.5–1.5 M betaine supplementation have been used to increase polymerase activity through Mg^2+^ availability and stabilized melting temperature of CG-rich DNA targets, respectively (Foo et al., 2020; Özay and McCalla, 2021; Rees et al., 1993). Ready-to-use reaction mixes and next-generation DNA polymerases can increase polymerization speed, thermal stability, and tolerance to inhibitory compounds (Yang et al., 2024). Most primer sets evaluated here were previously described using customized reaction mixes. We used a harmonized protocol for all 14 LAMP assays for comparative evaluation, including an optimized isothermal master mix ISO-004 (OptiGene Ltd.) containing a proprietary GspSSD2.0 DNA polymerase, MgSO_4_, dNTP, and ds-DNA binding dye. This kit and primer concentrations/ratios (0.1 μM F3/B3, 1.8 μM FIP/BIP, and 1 μM LF/LB) were demonstrated previously to be robust and optimum, and recommended for foodborne pathogen detection in food (Chen et al., 2011; Domesle et al., 2022; Ge et al., 2026). Because small variations of LAMP reagents yielded different assay outcomes (Foo et al., 2020; Yeh et al., 2005), the use of a universal master mix could have affected the performance of some of the LAMP assays.

Variations in LAMP reaction conditions (e.g., temperature profile, time), platforms (e.g., heating block, thermocycler), detection chemistries (i.e., turbidity, real-time fluorescence) also affect amplification efficiency and outcome. All LAMP assays in this study were carried out for comparative evaluation for isothermal amplification at 65°C for 30 min with real-time fluorescence monitoring and detection based on a stringent peak ratio threshold (T_p_ cutoff at 20 min), as recommended by the Genie II manufacturer based on ISO-004 kit performance and used similarly by the current *Salmonella* LAMP protocol in BAM (Andrews et al., 2026). Notably, the reaction conditions and cutoff values were different from those reported originally for these 14 *hlyA*-based assays. For instance, the FDA-2026 assay adopted a T_p_ cutoff of 30 min (Ge et al., 2026), while even longer reaction times were used in other assays. Primer sets that failed to detect *L. monocytogenes* (Tang-2011, Shan-2012, and Wang-2012) in this study were all previously evaluated with longer reaction time than 30 min (Shan et al., 2012; Tang et al., 2011; Wang et al., 2012b). Other primer sets that reported the same or lower LODs were also achieved using longer reaction time, as long as 65 min (Ma et al., 2019). However, extended periods of amplification increase the chance of unspecific LAMP amplicons, greatly compromising specificity (Jainonthee et al., 2022; Shan et al., 2012; Urrutia-Cabrera et al., 2021). Since LAMP assays are highly sensitive, carry-over environmental DNA or cross-contamination between samples can add unintended targets to the reaction and contribute to false-positive results (Hsieh et al., 2014; Suleman et al., 2016). Therefore, using a stringent T_p_ cutoff is desired to ensure efficient detection with low false-positive rates.

The evaluation of quantification capability showed varied results among the 14 LAMP assays (Figure 4). A strong quantification capability is important for extending the assay beyond presence/absence, especially when the target is present at high concentrations in certain food samples, and culture enrichment is not needed. Although various LAMP reaction conditions and detection chemistries present distinct advantages and disadvantages, LAMP remains a highly rapid, sensitive, and specific nucleic acid amplification technique and is promising for *L. monocytogenes* detection in various food applications.

Finally, we emphasize that a harmonized LAMP protocol was used in this study, not those individually reported/optimized conditions. Although this allowed for fair comparisons, it may not have represented the optimal performance of each assay. Nonetheless, under the harmonized protocol performed here, the recently reported FDA/CVM assay (Ge et al., 2026) had the best overall performance. As noted in the original report (Ge et al., 2026), the LAMP assay, upon further validations, may be used in food testing laboratories for routine screening of *L. monocytogenes* and used in conjunction with BAM culture to ensure product safety and protect public health.

## Conclusions

This work provides an independent comparative evaluation of 14 *hlyA*-based *L. monocytogenes* LAMP primer sets on the assays' sensitivity, specificity, and quantification capability. While we acknowledge the limitation of using a harmonized yet robust LAMP protocol for comparative analyses of all assays, it is apparent that “under the conditions used in the present study” the recently reported FDA/CVM assay (Ge et al., 2026) outperformed the 13 other primer sets. Additionally, primer sets Ye-2015 and Huang-2015 excelled in rapidity, primer sets Huang-2015 and Rivas-Macho-2023 outperformed in inclusivity, primer sets Ye-2015, Huang-2015, Huang-2015, and Rivas-Macho 2023 in sensitivity, and primer sets Wang-2011, Huang-2015, and Ye-2015 in quantification capability. Given these varied performances among the 14 LAMP assays evaluated, the present study highlights the practical importance of the standardization and selection of LAMP assays in food safety laboratories. It is important to emphasize that further validations are needed in real food matrices and under routine laboratory conditions for many of the assays, e.g., the validation of the FDA LAMP assay in soft cheese and raw pet food was reported (Ge et al., 2026). Our findings contribute to the integration of LAMP as an effective screening tool into the *L. monocytogenes* testing workflow in food for active pathogen surveillance and outbreak investigations.

## Data Availability

The datasets presented in this study are included in the article and further inquiries can be directed to the corresponding authors.
